# Cardiovascular disease threat and perceived efficacy of selected preventive behaviors among Polish men: an analysis based on the extended parallel process model

**DOI:** 10.3389/fpubh.2023.1244302

**Published:** 2023-11-13

**Authors:** Katarzyna Domosławska-Żylińska, Dorota Włodarczyk, Magdalena Krysińska-Pisarek

**Affiliations:** ^1^Department of Education and Communication, National Institute of Public Health NIH – National Research Institute, Warsaw, Poland; ^2^Department of Health Psychology, Medical University of Warsaw, Warsaw, Poland

**Keywords:** CVD, communication, prevention, EPPM, public health

## Abstract

Considering the low rate of preventive behaviors (5%), it is important to increase the effectiveness of actions that motivate the public to engage in health-promoting behaviors. The purpose of this study was to assess the way in which Polish men perceive the threat of cardiovascular diseases (CVDs) and the effectiveness of five preventive behaviors in the context of CVDs. We aimed to identify groups of recipients, based on the extended parallel process model (EPPM), for five preventive behaviors and to compare the identified groups in terms of selected characteristics. We conducted the survey in November 2022, using the computer-assisted web interviewing technique, on a representative sample of 1,000 men aged 18–65 years. Polish men showed relatively low levels of perceived susceptibility to CVDs (15.1%), but at the same time tended to perceive the consequences of CVDs as severe (54.2%). Segmentation of audiences according to the EPPM showed that regardless of the type of preventive behavior, the most numerous groups are responsive (31–37%) and indifferent (29–31%). This study revealed the need to increase awareness of the importance of a healthy diet to prevent CVDs among male population. Less than half of the men indicated that they would be able to implement effective stress management (49.8%) and smoking avoidance (39.4%), indicating the need to implement measures to increase self-efficacy in the areas.

## 1. Introduction

Cardiovascular diseases (CVDs) are the leading cause of death worldwide. In 2019, an estimated 17.9 million people died from CVDs, accounting for 32% of all deaths (1). In Poland, CVDs accounted for 39.4% of all deaths. More than four out of five CVD-related deaths result from heart attacks and strokes (2). Deaths caused by CVDs are more common in men than in women. The most important preventive behaviors for CVDs include: avoiding the use of tobacco products, adhering to a healthy diet, regular physical activity, maintaining a healthy body weight, limiting alcohol intake, introducing stress management strategies, maintaining normal blood pressure, and maintaining a normal range of low-density lipoprotein cholesterol (LDL-C) and non-high-density lipoprotein cholesterol (non-HDL-C) levels (3–6). The American Heart Association estimates that only 5% of people adhere to all of the above lifestyle recommendations (3). Considering the low level of preventive behavior, it is important to increase the effectiveness of actions aimed at motivating the public to engage in health-promoting behaviors.

A number of theoretical models have been developed to identify factors driving health behaviors (7). They include various aspects of threat and efficacy, the impact of which on lifestyle change has been confirmed in several studies (8, 9). These two factors and the relationships between them are the essence of the extended parallel process model (EPPM). According to this model, the combination of different levels of threat and efficacy determine the probability of behavioral implementation. On the one hand, people who estimate that they have an increased risk of CVDs are more likely to adopt a healthy lifestyle (10–12). On the other hand, threat estimation is associated with the optimistic bias—that is, the tendency to view one’s individual threat as lower than the actual threat (absolute threat) or lower than in others in a similar threat group (comparative threat) (13). However, the effects of perceived threat are different depending on the level of perceived efficacy. Different aspects of efficacy can be considered. It may refer to expectations about the outcome of an action (e.g., positive or negative) (14). Another option is to focus on self-efficacy: the belief that an individual is able to take and continue, despite obstacles, an action that will lead to a certain outcome (14). According to the EPPM, perceived efficacy coupled with a perceived threat of a given health problem is essential to implement targeted health behavior (15–17). Four variables are included in the EPPM model: Two of them comprise the perceived threat dimension (perceived susceptibility and perceived severity) and two comprise the perceived efficacy dimension (response efficacy and self-efficacy). According to the EPPM, based on these two dimensions, four groups of recipients can be identified: indifferent (low threat, low efficacy), avoidant (high threat, low efficacy), proactive (low threat, high efficacy), and responsive (high threat, high efficacy). Each group requires a different communication style and emphasis of different knowledge areas.

The EPPM has been used in health promotion for many health problems, such as HIV/AIDS, lung cancer, colorectal cancer, avian flu, breast self-examination, and smoking (18–23). The high prevalence of CVDs in Poland and its serious consequences constitute a rationale for the use of the EPPM as the basis for a health-promotion campaign. Data assessing the perception of threat and the effectiveness of implementing a healthy lifestyle in the context of CVD prevention are very limited in Poland. The issue is particularly relevant among men, who participate in prevention programs less often than women (24, 25).

The purpose of the study was: (a) to assess how Polish men perceive the threat of CVDs and the efficacy of five CVD preventive behaviors; (b) based on the EPPM, to classify the participants into four specific groups for the five preventive behaviors, namely a healthy diet, regular physical activity, limiting tobacco use, stress management, and regular examinations according to medical recommendations; and (c) to compare the groups according to selected characteristics, especially declared frequency of analyzed behaviors, perceptions of losses associated with these behaviors, and basic sociodemographic factors.

## 2. Materials and methods

The survey was conducted in November 2022, using the computer-assisted web interviewing technique, on a representative sample of 1,000 men (Table 1). The selection of respondents met the following assumptions:

- The number of people in the sample in each voivodeship was proportional to the number of inhabitants in the voivodeship;
- The proportion of age groups (18–29, 30–39, 40–59, and 60–65 years) was maintained in each voivodeship;
- The proportion of urban and rural inhabitants by age groups (18–29, 30–39, 40–59, and 60–65 years) was maintained across the country.

**Table 1:** Flow chart of the study.

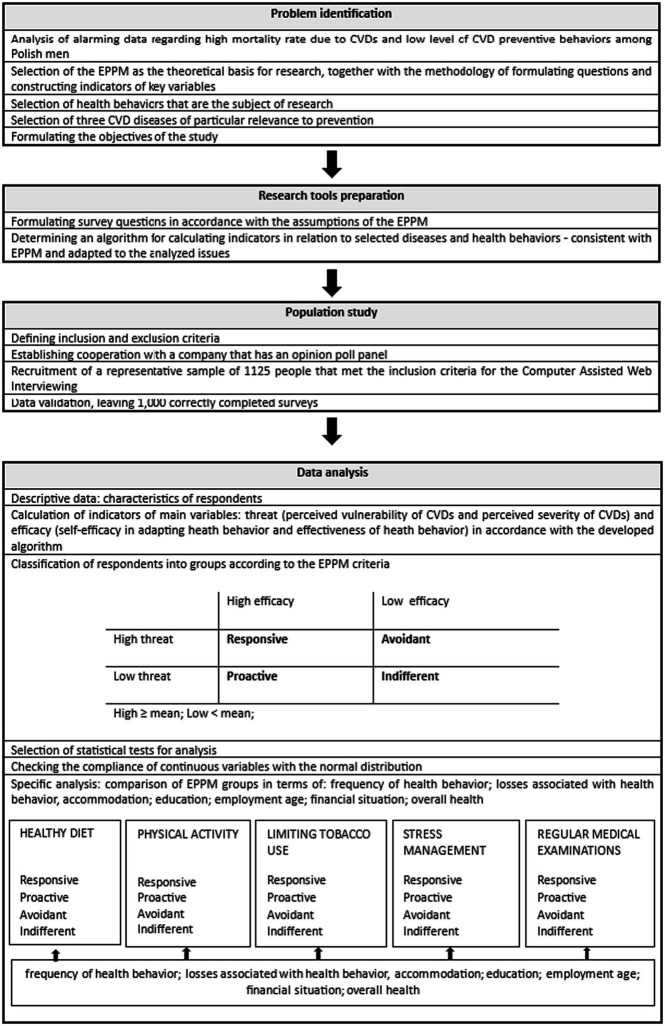

The inclusion criteria were: sex (male), age (adults 18–65 years from the general, non-clinical population), and consent to participate in the study. There were no exclusion criteria. The survey tool was an opinion poll panel. Participation in the survey was voluntary and anonymous. Invitations to the survey were sent to randomly selected users. Sociodemographic data were verified using the survey’s inclusion (metric) questions. The survey participants were informed about possibility to terminate the study at any point. The survey met the guidelines for protecting individuals in terms of their security and privacy.

The survey consisted of four parts. The first part focused on sociodemographic data. The second part included questions about declared frequency of implementation of the five preventive behaviors under study: a healthy diet, regular physical activity, avoidance of smoking, effective stress management, and carrying out medical examinations as recommended. The respondents rated the frequency of these behaviors on a 5-point scale, from 1 (almost never or never) to 5 (daily/always), with reverse scoring for tobacco smoking. The third part was designed to identify the EPPM groups, based on CVD threat perception and the efficacy of CVD preventive behaviors. Two questions were used to measure CVD threat perception: “How likely is it that you develop a specific medical condition (atherosclerosis, stroke, and myocardial infarction included in separate questions) at some point in the future?” (perceived vulnerability) and “How serious/harmful would be the consequences (physical or personal) of this disease?” (perceived severity evaluated for each disease separately). To measure perceived efficacy, the following questions were asked (separately for each preventive behavior): “In your opinion, how effective is the behavior in reducing the risk of the conditions listed?” (behavioral efficacy) and “How do you assess your ability to implement this behavior to reduce the risk of the following diseases?” (self-efficacy). The measurement tool was a 5-point Likert scale. The fourth part of the survey assessed the losses that the respondents would potentially incur as a result of implementing each of the preventive behaviors. The responses were rated on a 5-point Likert scale.

A threat indicator and an efficacy indicator were used to classify the respondents into the EPPM groups. This efficacy indicator (calculated for each preventive behavior separately) is the mean of the responses to the two questions regarding behavioral efficacy and self-efficacy. Similarly, the threat indicator is the mean of the responses regarding perceived susceptibility and perceived severity. Next, by selecting low (below the mean) and high (above the mean) levels of perceived efficacy and perceived threat, four EPPM groups were created. Differences among the groups were determined with one-way analysis of variance (ANOVA) or the chi-square (χ^**2**^) test, depending on the measurement scales. Microsoft Excel and IBM SPSS were used for calculations.

## 3. Results

### 3.1. Characteristics of the respondents

The study included 1,000 Polish male citizens, aged 18–65 years, with an average age of 41.8 years (Table 2). The majority of the study participants were working men (81.7%), with secondary or higher education (82.6%), and a self-assessed average financial situation (57.6%). A large portion of the respondents (41.7%) lived in rural areas. Almost half (48.7%) of the respondents described their health as good or very good (Table 2). Less than half of the respondents consumed a healthy diet (39.6%) and performed physical activity (39.4%) at least three times a week. Of the respondents, 44% declared that they were non-smokers. The majority of the respondents (58.6%) reported that they often or almost always attended medical examinations in accordance with their doctors’ recommendations. Among the participants, 19.2% declared the ability to manage stress (answers often and very often) (Supplementary Table S1) Only 5.3% of the participants declared that they followed all of the recommendations aimed at reducing the risk of CVDs (answers “often” or “very often” for each health behavior).

**Table 2:** Characteristics of the respondents (*n* = 1,000).

		%
Age (years)	18–29	20.5
	30–39	24.8
	40–49	25.7
	50–59	17.1
	60–65	11.9
Accommodation	Countryside	41.7
	Town ≤200,000	29.0
	Town 200,000–500,000	9.1
	Town ≥500,000	20.2
Education	Elementary or junior high school	2.8
	Basic vocational	14.6
	Secondary or post-secondary	42.6
	Higher education	40.0
Employment	Employed (full-time or self-employed)	81.7
	Student	4.0
	Unemployed	4.3
	Pensioner/Retiree	9.4
	Household leader	0.6
Self-assessment of financial situation	Very bad	2.7
	Bad	10.0
	Average	57.6
	Good	25.9
	Very good	3.8
Self-assessment of overall health	Very bad	1.8
	Bad	8.8
	Average	40.7
	Good	41.8
	Very good	6.9

### 3.2. CVD threat and effectiveness of the five preventive behaviors

#### 3.2.1. Threat: perceived susceptibility and perceived severity

We assessed two factors to evaluate subjective perception of CVD threat: perceived CVD susceptibility (Supplementary Table S2) and perceived CVD severity (Supplementary Table S3). We created the overall CVD indicators as the means of the responses to three diseases: atherosclerosis, stroke, and myocardial infarction. We found that 15.1% of respondents considered developing CVDs as probable or very probable and 45.4% considered it as not very probable or improbable. The respondents considered the development of myocardial infarction to be the most likely (16.2%), followed by atherosclerosis and stroke (15.2 and 13.8%, respectively). More than half (54.2%) of the respondents believed that developing CVDs would be harmful or very harmful for them and would be associated with serious consequences. Only 12.2% of the respondents considered that it would be of minor or no harm. The most harmful consequences were assigned to atherosclerosis (55.8%), followed by myocardial infarction (54.0%) and stroke (52.7%).

#### 3.2.2. Efficacy: perceived efficacy of preventive behavior and perceived self-efficacy

We considered two factors to assess the effectiveness of the five recommended preventive behaviors: the perceived efficacy of each behavior (Supplementary Table S4) and self-efficacy of the implementation of each behavior (Supplementary Table S5). The respondents considered the health behaviors to be effective or very effective in preventing CVDs in the following order: avoiding smoking (63.1%), regular medical examinations (59.2%), physical activity (56.8%), effective stress management (55.9%), and a healthy diet (49.7%). The respondents believed that they could (answers “possible” or “very possible”) implement particular preventive behaviors in the following order: regular medical examinations (58.8%), physical activity (53.3%), healthy diet (53.0%), effective stress management (49.8%), and avoiding smoking (39.4%).

### 3.3. Identification of groups according to the EPPM for each preventive behavior and their comparison in terms of selected characteristics

We identified the four groups defined by the EPPM based on the combination of perceived CVD threat (high or low) and perceived efficacy (high or low) separately for each preventive behavior (with the mean of a given dimension as the criterion of division). For the threat dimension, the mean was 3.08. For efficacy, the means were as follows: 3.45 for a healthy diet, 3.57 for physical activity, 3.37 for smoking avoidance, 3.5 for stress management, and 3.65 for regular medical examinations.

#### 3.3.1. A healthy diet

Table 3 shows that among the EPPM groups, the indifferent and avoidant groups were less likely to eat healthily compared with the proactive and responsive groups. The avoidant group, relative to the proactive and responsive groups, had a higher loss rate regarding the implementation of regular healthy eating. In addition, the responsive group was older than the other groups. The indifferent group assessed their financial situation as worse compared with the proactive and responsive groups, and their health condition as worse compared with the responsive group (Table 3). The groups also differed in terms of the education level (Supplementary Table S6). In the responsive group, most men declared they had received higher education, while in the indifferent group, most men declared primary, junior high, or basic vocational education (χ^2^ = 21.99, *p* < 0.05).

**Table 3:** Differences between the groups according to the extended parallel process model (EPPM) in relation to a healthy diet as a cardiovascular disease preventive behavior.

Group	Indifferent (I) (*n* = 285)	Proactive (P) (*n* = 182)	Avoidant (A) (*n* = 170)	Responsive (R) (*n* = 363)	F (p)	*Post hoc*
Age (years)	40.5 (12.1)	41.1 (13.2)	40.7 (12.9)	43.7 (12.4)	4.47 (<0.05)	R > I R > A R > P
Financial situation	3.02 (0.824)	3.29 (0.717)	3.13 (0.710)	3.28 (0.746)	7.94 (<0.001)	P > I R > I
Overall health	3.27 (0.881)	3.48 (0.865)	3.39 (0.739)	3.55 (0.754)	6.71 (<0.001)	R > I
Frequency: a healthy diet	2.47 (1.194)	3.54 (1.129)	2.48 (1.173)	3.49 (1.171)	62.82 (<0.001)	P > I P > A R > I
Losses associated with the implementation of a healthy diet	2.81 (0.826)	2.69 (0.953)	3.02 (0.626)	2.73 (0.919)	5.71 (<0.001)	A > P A > R

The data are presented as the mean (standard deviation).

#### 3.3.2. Regular physical activity

Table 4 show that the frequency of regular physical activity in the responsive and proactive groups was higher compared with the indifferent group and/or responsive groups. The avoidant and/or indifferent groups perceived greater losses when implementing regular physical activity compared with the proactive group. Moreover, the indifferent and avoidant groups indicated a more difficult financial situation compared with the responsive group. The indifferent group assessed their health status as worse than the proactive and responsive groups (Table 4). The groups differed significantly in terms of the education level (Supplementary Table S7). In the responsive group, most men had a university degree, while in the indifferent group, most men declared primary, junior high, or basic vocational education (χ^2^ = 24.99, *p* < 0.05).

**Table 4:** Differences between the groups according to the extended parallel process model (EPPM) in relation to regular physical activity as a cardiovascular disease preventive behavior.

Group	Indifferent (I) (*n* = 303)	Proactive (P) (*n* = 164)	Avoidant (A) (*n* = 200)	Responsive (R) (*n* = 333)	F (p)	*Post hoc*
Financial situation	3.04 (0.833)	3.27 (0.695)	3.08 (0.645)	3.32 (0.774)	9.53 (<0.001)	R > I R > A
Overall health	3.25 (0.863)	3.54 (0.882)	3.39 (0.728)	3.57 (0.760)	9.42 (<0.001)	P > I R > I
Frequency: regular physical activity	2.78 (1.191)	3.57 (1.080)	2.85 (1.247)	3.41 (1.165)	26.536 (<0.001)	P > I R > I P > A
Losses associated with the implementation of regular physical activity	2.69 (0.851)	2.32 (1.08)	2.84 (0.787)	2.49 (1.008)	11.415 (<0.001)	I > P A > P A > R

The data are presented as the mean (standard deviation).

#### 3.3.3. Limiting tobacco use

Table 5 shows that the proactive and responsive groups were more likely to reduce tobacco use compared with the indifferent group. The avoidant group identified greater losses associated with implementing smoking reduction compared with the indifferent, responsive, and proactive groups. In addition, the proactive group was older than the indifferent group. The responsive, proactive, and avoidant groups described their financial situation as better compared with the indifferent group. The responsive group assessed their health as better compared with the indifferent group (Table 5). The groups differed significantly in terms of the employment status (χ^2^ = 24.258, *p* < 0.05). The responsive group had the highest percentage of employed and retired participants, while the indifferent group had the highest percentage of unemployed participants and housekeepers (Supplementary Table S8).

**Table 5:** Differences between the groups according to the extended parallel process model (EPPM) in relation to limiting tobacco use as a cardiovascular disease preventive behavior.

Group	Indifferent (I) (*n* = 173)	Proactive (P) (*n* = 115)	Avoidant (A) (*n* = 88)	Responsive (R) (*n* = 184)	F (p)	*Post hoc*
Age (years)	39.4 (11.8)	43.6 (12.6)	41.1 (12.6)	43.1 (12.7)	3.72 (<0.05)	P > I
Financial situation	2.99 (0.886)	3.22 (0.723)	3.22 (0.651)	3.23 (0.818)	3.26 (<0.05)	R > I A > I P > I
Overall health	3.22 (0.895)	3.37 (0.882)	3.47 (0.710)	3.51 (0.768)	4.05 (<0.05)	R > I A > I
Frequency: limiting tobacco use	3.25 (1.42)	2.82 (1.68)	2.99 (1.64)	2.82 (1.66)	2.78 (<0.05)	P > I R > I
Losses associated with limiting tobacco use	2.64 (0.831)	2.73 (1.09)	3.03 (0.724)	2.95 (1.12)	4.66 (<0.05)	A > I I > R A > P

The data are presented as the mean (standard deviation).

#### 3.3.4. Stress management

As presented in Table 6 the indifferent and avoidant groups controlled their emotions worse than the proactive and responsive groups. The indifferent group expected greater losses when implementing effective emotion control compared with the proactive and responsive groups. The indifferent and avoidant groups declared a worse financial situation compared with the responsive group (Table 6). The health status of the indifferent group was worse than the proactive and responsive groups. The groups differed significantly (χ^2^ = 24.93, *p* < 0.05) in terms of the education level (Supplementary Table S9). The indifferent group included significantly more respondents with primary, junior high, and basic vocational education than the proactive group.

**Table 6:** Differences between the groups according to the extended parallel process model (EPPM) in relation to stress management as a cardiovascular disease preventive behavior.

Group	Indifferent (I) (*n* = 296)	Proactive (P) (*n* = 171)	Avoidant (A) (*n* = 226)	Responsive (R) (*n* = 307)	F (p)	*Post hoc*
Age (years)	40.3 (12.2)	41.3 (13.1)	41.5 (12.1)	43.7 (12.9)	3.76 (<0.05)	R > I
Financial situation	3.06 (0.842)	3.23 (0.695)	3.10 (0.613)	3.33 (0.804)	7.41 (<0.001)	R > I R > A
Overall health	3.26 (0.884)	3.52 (0.849)	3.42 (0.697)	3.56 (0.787)	7.76 (<0.001)	P > I R > I
Frequency: stress management	3.05 (1.06)	3.80 (1.08)	3.26 (1.07)	3.71 (1.02)	28.901 (<0.001)	P > I R > I P > A R > A
Losses associated with the implementation of effective stress management techniques	2.60 (0.880)	2.41 (1.11)	2.49 (0.818)	2.39 (1.11)	2.67 (<0.05)	I > P I > R

The data are presented as the mean (standard deviation).

#### 3.3.5. Regular medical examinations

Table 7 shows that compared with the proactive and responsive groups, the indifferent and avoidant groups were less likely to attend regular check-ups. The avoidant group expected greater losses when implementing regular examinations compared with the proactive and responsive groups. Similarly, the indifferent group described their losses as greater compared with the responsive group. In addition, the indifferent group described their financial situation as worse than the proactive and responsive groups. The indifferent group had a worse health status than the responsive group (Table 7). The groups differed significantly (χ^2^ = 25.13, *p* < 0.05) in terms of the education level (Supplementary Table S10). The responsive group included significantly more respondents declaring higher education than the indifferent group.

**Table 7:** Differences between the groups according to the extended parallel process model (EPPM) in relation to regular medical examinations as a cardiovascular disease preventive behavior.

Group	Indifferent (I) (*n* = 297)	Proactive (P) (*n* = 170)	Avoidant (A) (*n* = 160)	Responsive (R) (*n* = 373)	F (p)	*Post hoc*
Age	40.3 (12.3)	41.4 (12.9)	41.5 (12.3)	43.3 (12.8)	3.10 (<0.05)	R > I
Financial situation	3.03 (0.838)	3.29 (0.683)	3.16 (0.643)	3.26 (0.773)	6.67 (<0.001)	P > I R > I
Overall health	3.27 (0.897)	3.51 (0.830)	3.46 (0.734)	3.52 (0.760)	6.13 (<0.001)	R > I
Frequency: regular medical examinations	2.61 (1.02)	3.38 (1.196)	2.80 (1.039)	3.44 (1.164)	38.261 (<0.001)	P > I R > I R > A
Losses associated with the implementation of regular medical examinations	2.70 (0.850)	2.46 (1.09)	2.88 (0.775)	2.40 (1.095)	11.390 (<0.001)	I > R A > P A > R

The data are presented as the mean (standard deviation).

## 4. Discussion

Analysis of threat variables has shown that Polish men have a relatively low sense of the occurrence of CVDs (perceived susceptibility). The prevalence of those indicating a low probability of risk may confirm the phenomenon of optimistic bias (or unrealistic optimism) (13). Simultaneously, the respondents showed a tendency to consider the consequences of CVDs as severe. The findings suggest the need to develop specific interventions to increase awareness of CVDs risk.

The respondents seemed to be convinced of the effectiveness of the CVD preventive behaviors analyzed in the study. They rated the effectiveness of a healthy diet relatively low, suggesting the need to increase men’s awareness of its importance in CVD prevention. The respondents declared quite high self-efficacy in implementing regular medical examinations, physical activity, and a healthy diet. Tobacco use avoidance is an example of something men are convinced is effective, but they are also aware of their own limitations in implementing the change. Therefore, preventive behaviors aimed at increasing self-efficacy to reduce tobacco use and effective addiction treatment, but also promotion of effective forms of stress management (e.g., workshops in the workplace), would be advisable (26).

The classification of respondents according to the EPPM revealed that regardless of the type of preventive behavior, the responsive and indifferent groups had the most respondents. These are completely opposite groups in terms of the threat and efficacy levels. This result seems consistent with the predictions of the EPPM and indicates an accurate classification in the current study (14).

We found that men in the responsive and proactive groups were more likely to implement CVD preventive behaviors compared with men in the indifferent group (regarding all analyzed behaviors) and in the avoidant group (selected behaviors). Although the responsive and proactive groups have a different threat level, they have a high efficacy level and presented a similarly high frequency for each preventive behavior. However, the proactive group had fewer respondents the responsive group.

It seems quite obvious that from a public health perspective (the frequency of health behavior implementation), the most desirable effect would be to increase the number of people in the responsive and proactive groups. We have demonstrated the beneficial role of efficacy, as defined by the EPPM, in the current study. However, it is still not clear why both high and low CVD threat levels result in similarly high levels of declared behaviors. Regarding the indifferent and avoidant groups, we can see that low efficacy levels resulted in lower frequency of the preventive behaviors in men with high and low threat levels. Thus, the following question arises: What aspects and intensity of CVD threat are likely to activate health behaviors? Other issue refers to the proactive group: What conditions must be met to develop CVD preventive behaviors in men with no or low awareness of CVD threats (but with high efficacy)? The process of developing healthy habits should probably start in early childhood, but this specific group requires further investigation.

The existing studies rarely refer to models of behavioral change, including the EPPM. A Polish study of the determinants of positive health behaviors among men found that a good financial situation, positive views of work and life, high self-esteem of health care, and male psychological gender were the most conducive to taking care of health (27). On the other hand, blue-collar workers, characterized by a low education level and low health awareness, require intervention in the area of health education. In contrast, an Australian study found that the determinants of the use of dedicated health services for men are primarily declared health problems and motivation to change their health (28).

By adopting the EPPM, we achieved a more thorough differentiation of the recipients of health promotion. Looking for the factors differentiating the groups, we found that the responsive group differed significantly from the indifferent group in terms of the financial situation and self-assessed health status in all five analyzed behaviors. The responsive group rated their financial situation and health status as better than men in the indifferent group. This shows that health and financial problems can be serious obstacles in the implementation of desired health behaviors. Studies have confirmed the relationship between a worse financial situation and the occurrence of CVDs (29, 30). Additionally, individuals who are more exposed to health risks have lower motivation to engage in health-promoting behaviors (31). Such low motivation may result, for example, from the belief that the costs of engaging in a particular behavior outweigh its benefits. This may contribute to a lack of commitment to health-promoting activities due to a belief that such activities are of low value and importance in the current situation (32–34). However, from a long-term perspective, that kind of perception is harmful. Strategies aimed at increasing motivation should focus on the decision-making balance and on changing beliefs about the necessary effort or values regarding health behaviors (35).

The responsive group was significantly older than the indifferent group in three analyzed behaviors (a healthy diet, stress management, and regular medical examinations). Regarding a healthy diet, the responsive group was also older than the proactive group. This may confirm that the perception of health risks increases with aging, which in turn may result in an increased interest in health issues and a willingness to take care of one’s health. This direction should be reflected in the development of health information messages. In four of the five preventive behaviors, the groups differed significantly in terms of the education level. The responsive group included more men declaring higher education, while the indifferent group was dominated by men with primary, junior high, or basic vocational education. Other studies have confirmed the need to implement strategies aimed at reducing risk factors and promoting preventive behaviors targeting less educated people due to their higher risk of death from CVDs (36, 37). A lower education level and socioeconomic status are associated with insufficient implementation of CVD preventive behaviors. Primary care physicians can play a key role in counteracting this issue by, for example, encouraging health-promoting behaviors (29). Increasing health literacy among groups that are most at risk seems to be essential: Educational activities effectively increase motivation to implement CVD preventive behaviors (38).

An added value of our study is the analysis of losses possibly related to the implementation of each preventive behavior. The indifferent group rated their losses (regarding limiting tobacco use, stress management, and regular medical examinations) as greater compared with the responsive group. The avoidant group described their losses (except for stress management) as significantly greater compared with the proactive group. We noted that men with a low efficacy component described greater losses (indifferent group vs. responsive group and proactive group vs. avoidant group).

In summary, based on our results, it seems that the key component influencing the differences between the responsive and proactive groups versus the indifferent group is efficacy (motivation). Duffy et al. (39) also highlighted this element when they conceptualized that motivation increases self-efficacy regarding the application of CVD preventive behaviors combined with available knowledge about the effectiveness of these behaviors. These authors suggest focusing on the motivation factor to achieve the expected CVD prevention outcomes. However, adhering to a healthy lifestyle in the long term is an area that requires further investigation. Duffy et al. (39) suggest increasing competence in applying effective motivation among medical personnel (doctors and nurses) responsible for patient education. This would ensure an individualized approach to a patient focusing on discussing key motivational factors (emotional, cognitive, economic, etc.) for CVD prevention. The effectiveness of this approach has been confirmed by Project Leonardo carried out in 2006–2007 in Italy. This project examined the effectiveness of a care manager role in the health care system to support primary care physicians and specialists in the treatment of patients with CVDs, diabetes, or heart failure. Care managers worked directly with individual patients on education and motivation. With a care manager, there was better adherence to the recommendations, improved clinical parameters, and better disease control (40).

The current study is exploratory and as such has some limitations. Although there are some benefits to using a survey, there are numerous weaknesses. One of them is the declarative character of the data. This is especially important for the declared frequency of healthy behaviors, which can be distorted by social desirability. The respondents may not feel comfortable providing responses that present them in an unfavorable light. Subjectivity in the interpretations of survey response options (like frequencies) may contribute to answer inconsistency. We also based the EPPM classification on the subjective assessment of the respondents; therefore, the assignment to groups might be distorted. Adoption of newer technologies, such as mobile applications or wearable devices, might increase measurement accuracy (41). Even knowing that in 2022 93.3% of households in Poland had access to the Internet (broadband fixed or mobile), it is also necessary to consider the risk of accessibility bias to computer equipment and the Internet, and/or computer skills. Future research on health behavior should involve more advanced methods to reduce the risk of under– or overreports. Other aspects of the study like self-efficacy or threat perception may also be biased by defense mechanisms or defense optimism. Additionally, we have included only three of the most representative CVDs, and they certainly do not encompass the entire range of this medical problem. Finally, this is one of the first studies testing the EPPM in the Polish population. Including more factors would allow for more in-depth characterization of the groups and detection of more specific differences between the responsive and proactive groups, and between the indifferent and avoidant groups. Future research should include an in-depth analysis of the identified EPPM groups.

In conclusion, we have shown that among Polish men efforts are needed to increase both self-efficacy and perceived effectiveness of CVD preventive health behaviors. Further research is needed to explain the role of perceived threat and the mutual relationships between the susceptibility and severity of specific CVDs.

## Data availability statement

The raw data supporting the conclusions of this article will be made available by the authors, without undue reservation.

## Ethics statement

Ethical approval was not required for the studies involving humans because during the period of the survey, according to local law. The studies were conducted in accordance with the local legislation and institutional requirements. The participants provided their written informed consent to participate in this study.

## Author contributions

KD-Ż: study conception, developed the theoretical framework, data collection, and manuscript draft preparation. DW: study design, analysis and interpretation of results, and supervised the manuscript. MK-P: writing – original draft, funding acquisition, formal analysis, and project administration. All authors contributed to the article and approved the submitted version.

## References

[1] WHO. (n.d.) Cardiovascular diseases (CVDs). Available at: https://www.who.int/news-room/fact-sheets/detail/cardiovascular-diseases-(cvds) (Accessed January 19, 2023).

[2] NIPH NIH-NRI. Sytuacja zdrowotna ludności Polski i jej uwarunkowania (2022). Available at: http://bc.gbpizs.gov.pl/publication/7275/edition/7140/sytuacja-zdrowotna-ludnosci-polski-i-jej-uwarunkowania-2022?language=en (Accessed January 19, 2023).

[3] Rippe JM. Lifestyle strategies for risk factor reduction, prevention, and treatment of cardiovascular disease. Am J Lifestyle Med. (2019) 13:204–12. doi: 10.1177/1559827618812395, PMID: 30800027 PMC6378495

[4] Teo KK Rafiq T. Cardiovascular risk factors and prevention: a perspective from developing countries. Can J Cardiol. (2021) 37:733–43. doi: 10.1016/j.cjca.2021.02.009, PMID: 33610690

[5] Dobrosielski DA. How can exercise reduce cardiovascular disease risk? A primer for the clinician. Pol. Arch Intern Med. (2021) 131:16122. doi: 10.20452/pamw.16122 34706491

[6] Chinnaiyan KM. Role of stress management for cardiovascular disease prevention. Curr Opin Cardiol. (2019) 34:531–5. doi: 10.1097/HCO.0000000000000649 31219875

[7] Lerner JS Li Y Valdesolo P Kassam KS. Emotion and decision making. Annu Rev Psychol. (2015) 66:799–823. doi: 10.1146/annurev-psych-010213-115043 25251484

[8] Mehiriz K Gosselin P. The effect of perceived threats and response efficacy on adaptation to smog: an instrumental variables design. Risk Anal. (2022) 42:1042–55. doi: 10.1111/risa.13814, PMID: 34424564

[9] Affendi I Nor Asiah M Normi M Mohd Hatta AM Noor Aliza L Md Sabtuah MR. Association between self-efficacy and health behaviour in disease control: a systematic review. Glob J Health Sci. (2018) 10:18. doi: 10.5539/gjhs.v10n1p18

[10] Sheeran P Harris PR Epton T. Does heightening risk appraisals change people’s intentions and behavior? A meta-analysis of experimental studies. Psychol Bull. (2014) 140:511–43. doi: 10.1037/a0033065, PMID: 23731175

[11] Homko CJ Santamore WP Zamora L Shirk G Gaughan J Cross R. Cardiovascular disease knowledge and risk perception among underserved individuals at increased risk of cardiovascular disease. J Cardiovasc Nurs. (2008) 23:332–7. doi: 10.1097/01.JCN.0000317432.44586.aa 18596496

[12] Lynch EB Liu K Kiefe CI Greenland P. Cardiovascular disease risk factor knowledge in young adults and 10-year change in risk factors: the coronary artery risk development in young adults (CARDIA) study. Am J Epidemiol. (2006) 164:1171–9. doi: 10.1093/aje/kwj334, PMID: 17038418

[13] Bajada CJ. The optimism bias: a cognitive neuroscience perspective. Xjenza Online. (2004) 2:33–7. doi: 10.7423/XJENZA.2014.1.04

[14] Witte K. Putting the fear back into fear appeals: the extended parallel process model. Commun Monogr. (1992) 59:329–49. doi: 10.1080/03637759209376276

[15] Liu Q Huang Y-J Zhao L Wang W Liu S He G-P. Association between knowledge and risk for cardiovascular disease among older adults: a cross-sectional study in China. Int J Nurs Sci. (2020) 7:184–90. doi: 10.1016/j.ijnss.2020.03.008 32685615 PMC7355188

[16] Albarqouni L Smenes K Meinertz T Schunkert H Fang X Ronel J. Patients’ knowledge about symptoms and adequate behaviour during acute myocardial infarction and its impact on delay time: findings from the multicentre MEDEA study. Patient Educ Couns. (2016) 99:1845–51. doi: 10.1016/j.pec.2016.06.007, PMID: 27387122

[17] Hassen HY Bowyer M Gibson L Abrams S Bastiaens H. Level of cardiovascular disease knowledge, risk perception and intention towards healthy lifestyle and socioeconomic disparities among adults in vulnerable communities of Belgium and England. BMC Public Health. (2022) 22:197. doi: 10.1186/s12889-022-12608-z, PMID: 35093056 PMC8800212

[18] Afenigus AD Mulugeta H Tsehay B Gedfew M Ayenew T Getnet A. Behavioral response to HIV/AIDS prevention messages among students in selected universities of Amhara region, Northwest Ethiopia: an extended parallel process model. HIV AIDS. (2021) 13:115–24. doi: 10.2147/HIV.S288297 PMC7866924 33564268

[19] Zarghami F Allahverdipour H Jafarabadi MA. Extended parallel process model (EPPM) in evaluating lung Cancer risk perception among older smokers. BMC Public Health. (2021) 21:1872. doi: 10.1186/s12889-021-11896-1, PMID: 34657617 PMC8520616

[20] Nguyen AT. EPPM and its effectiveness in advertisements of colorectal cancer screening among young adults. Tampa (FL): USF (2020).

[21] Munch H. Avian and pandemic influenza knowledge and risk perception in southern Minnesota. Mankato (MN): Minnesota State University (2017).

[22] Chen L Yang X. Using EPPM to evaluate the effectiveness of fear appeal messages across different media outlets to increase the intention of breast self-examination among Chinese women. Health Commun. (2019) 34:1369–76. doi: 10.1080/10410236.2018.1493416 30080982

[23] Hosseini Z Mouseli A Aghamolaei TT Mohseni S Shahini S Dadipoor S. Predictors of adopting smoking preventive behaviors by university students: the extended parallel process model fitness test. J Subst Use. (2022) 1–8. doi: 10.1080/14659891.2022.2120423

[24] Jackson MC Dai S Skeete RA Owens-Gary M Cannon MJ Smith BD. An examination of gender differences in the National Diabetes Prevention Program’s lifestyle change program. Diabetes Educ. (2020) 46:580–6. doi: 10.1177/0145721720964585 33063641 PMC7802597

[25] Smith ML Bergeron CD Ahn S Towne SD Jr Mingo CA Robinson KT. Engaging the underrepresented sex: male participation in chronic disease self-management education (CDSME) programs. Am J Mens Health. (2018) 12:935–43. doi: 10.1177/1557988317750943, PMID: 29355070 PMC6131430

[26] WHO. Strengthening health systems for treating tobacco dependence in primary care / Part III. (2021). Available at: https://apps.who.int/iris/bitstream/handle/10665/84388/9789241505413_eng_Part-III_service_providers.pdf (Accessed May 25, 2023).

[27] Hildt-Ciupińska K Pawłowska-Cyprysiak K. Positive health behaviors and their determinants among men active on the labor market in Poland. Am J Mens Health. (2020) 14:1557988319899236. doi: 10.1177/1557988319899236, PMID: 32003283 PMC7099675

[28] Vincent AD Drioli-Phillips PG Le J Cusack L Schultz TJ McGee MA. Health behaviours of Australian men and the likelihood of attending a dedicated men’s health service. BMC Public Health. (2018) 18:1078. doi: 10.1186/s12889-018-5992-6, PMID: 30165836 PMC6117954

[29] Schultz WM Kelli HM Lisko JC Varghese T Shen J Sandesara P. Socioeconomic status and cardiovascular outcomes. Circulation. (2018) 137:2166–78. doi: 10.1161/CIRCULATIONAHA.117.029652, PMID: 29760227 PMC5958918

[30] Clark AM DesMeules M Luo W Duncan AS Wielgosz A. Socioeconomic status and cardiovascular disease: risks and implications for care. Nat Rev Cardiol. (2009) 6:712–22. doi: 10.1038/nrcardio.2009.163 19770848

[31] Sarah J Hancox J Hattar A Maxwell-Smith C Thøgersen-Ntoumani C Hagger MS. Motivating the unmotivated: how can health behavior be changed in those unwilling to change? Front Psychol. (2015) 6:835. doi: 10.3389/fpsyg.2015.00835 26136716 PMC4468355

[32] Vlachopoulous SP Gigoudi MA. Why don’t you exercise? Development of the amotivation toward exercise scale among older inactive adults. J Aging Phys Act. (2008) 16:316–41. doi: 10.1123/japa.16.3.316, PMID: 18660553

[33] Shen B Wingert RK Li W Sun H Rukavina PB. An amotivation model in physical education. J Teach Phys Educ. (2010) 29:72–84. doi: 10.1123/jtpe.29.1.72

[34] Wigfield A Eccles JS. Expectancy-value theory of achievement motivation. Contemp Educ Psychol. (2000) 25:68–81. doi: 10.1006/ceps.1999.1015 10620382

[35] Miller WR Rollnick S. Motivational interviewing: Preparing people for change. 3rd ed. New York, NY: Guildford Press (2013).

[36] Petrelli A Sebastiani G Di Napoli A Macciotta A Di Filippo P Strippoli E. Education inequalities in cardiovascular and coronary heart disease in Italy and the role of behavioral and biological risk factors. Nutr Metab Cardiovasc Dis. (2022) 32:918–28. doi: 10.1016/j.numecd.2021.10.022 35067447

[37] Carter AR Gill D Davies NM Taylor AE Tillmann T Vaucher J. Understanding the consequences of education inequality on cardiovascular disease: mendelian randomisation study. BMJ. (2019) 365:l1855. doi: 10.1136/bmj.l1855, PMID: 31122926 PMC6529852

[38] Navar AM Stone NJ Martin SS. What to say and how to say it: effective communication for cardiovascular disease prevention. Curr Opin Cardiol. (2016) 31:537–44. doi: 10.1097/HCO.0000000000000322, PMID: 27428113 PMC5045897

[39] Duffy EY Ashen D Blumenthal RS Davis DM Gulati M Blaha MJ. Communication approaches to enhance patient motivation and adherence in cardiovascular disease prevention. Clin Cardiol. (2021) 44:1199–207. doi: 10.1002/clc.23555 34414588 PMC8427972

[40] Ciccone MM Aquilino A Cortese F Scicchitano P Sassara M Mola E. Feasibility and effectiveness of a disease and care management model in the primary health care system for patients with heart failure and diabetes (project Leonardo). Vasc Health Risk Manag. (2010) 6:297–305. doi: 10.2147/vhrm.s9252 20479952 PMC2868351

[41] Żarnowski A Jankowski M Gujski M. Use of mobile apps and wearables to monitor diet, weight, and physical activity: a cross-sectional survey of adults in Poland. Med Sci Monit. (2022) 28:e937948. doi: 10.12659/MSM.937948 36081328 PMC9473310

