# Supplementary material for: Cardiovascular disease threat and perceived efficacy of selected preventive behaviors among Polish men: an analysis based on the extended parallel process model
Source: Front Public Health. 2023 Nov 13;11:1244302. doi: 10.3389/fpubh.2023.1244302 (PMC10679673; doi:10.3389/fpubh.2023.1244302)
Supplement: Supplementary file 1 [file Table_1.DOCX]

SUPPLEMENTARY

Table S1 The frequency of health behaviors (N=1000; %)

|  | Almost never or never | 1-2 times a month | 1-2 times a week | 3-4 times a week | everyday |
| --- | --- | --- | --- | --- | --- |
| Healthy diet | 16.8 | 16.9 | 26.7 | 25.0 | 14.6 |
| Regular physical activity | 11.9 | 17.7 | 31.0 | 24.1 | 15.3 |
| Cigarette smoking * | 15.4 | 7.6 | 10.4 | 5.6 | 17.0 |
|  | Almost never or never | rarely | occasionally | often | almost always or always |
| Regular medical examinations | 9.7 | 15.7 | 16.0 | 19.8 | 38.8 |
| Stress management | 31.6 | 31.4 | 17.8 | 13.8 | 5.4 |
| * Reverse scoring : Almost never or never (5); 1-2 times a month (4); 1-2 times a week (3); 3-4 times a week (2); everyday (1)  N=560 | | | | | |

Table S2 Probability of CVDs (n=1000; %)

|  | Improbable | Slightly probable | Moderately probable | Probable | Very probable |
| --- | --- | --- | --- | --- | --- |
| Atherosclerosis | 17.4 | 31.8 | 35.6 | **12.0** | **3.2** |
| Stroke | 14.0 | 31.2 | 41.0 | **10.6** | **3.2** |
| Myocardial infarction | 13.1 | 28.8 | 41.9 | **12.7** | **3.5** |
| Total CVDs | 14.8 | 30.6 | 39.5 | **11.8** | **3.3** |

Table S3 Perceived severity of CVDs (n=1000;%)

|  | Harmless | Slightly harmful | Moderately harmful | Harmful | Very harmful |
| --- | --- | --- | --- | --- | --- |
| Atherosclerosis | 0.8 | 12.1 | 31.3 | **39.5** | **16.3** |
| Stroke | 0.9 | 11.7 | 34.7 | **37.3** | **15.4** |
| Myocardial infarction | 0.7 | 10.3 | 35.0 | **37.8** | **16.2** |
| Total CVDs | 0.8 | 11.4 | 33.6 | **38.2** | **16.0** |

Table S4 Effectiveness of preventive behaviours, answer to the question: In your opinion, how effective is the behavior in reducing the risk of the conditions listed (n=1000; %)

|  |  | Ineffective | Not very effective | Moderately effective | Effective | Very effective |
| --- | --- | --- | --- | --- | --- | --- |
| Healthy diet | Atherosclerosis | 5.6 | 8.8 | 32.0 | 38.4 | 15.2 |
|  | Stroke | 5.7 | 11.7 | 37.3 | 33.3 | 12.0 |
|  | Myocardial infarction | 5.2 | 9.9 | 34.7 | 35.8 | 14.4 |
|  | CVDs | 5.5 | 10.1 | 34.7 | **35.8** | **13.9** |
| Regular physical activity | Atherosclerosis | 4.3 | 7.8 | 29.9 | 36.1 | 21.9 |
|  | Stroke | 4.9 | 9.2 | 32.5 | 33.9 | 19.5 |
|  | Myocardial infarction | 4.5 | 6.8 | 29.6 | 37.4 | 21.7 |
|  | CVDs | 4.6 | 7.9 | 30.7 | **35.8** | **21.0** |
| Avoidance of smoking | Atherosclerosis | 5.4 | 7.9 | 24.8 | 30.9 | 31.0 |
|  | Stroke | 5.2 | 7.8 | 23.9 | 32.3 | 30.8 |
|  | Myocardial infarction | 4.9 | 7.0 | 23.8 | 30.1 | 34.2 |
|  | CVDs | 5.2 | 7.6 | 24.1 | **31.1** | **32.0** |
| Effective stress management | Atherosclerosis | 5.7 | 13.1 | 33.7 | 30.0 | 17.5 |
|  | Stroke | 4.7 | 8.8 | 29.5 | 34.1 | 22.9 |
|  | Myocardial infarction | 3.4 | 7.0 | 26.6 | 35.1 | 27.9 |
|  | CVDs | 4.6 | 9.6 | 29.9 | **33.1** | **22.8** |
| Medical examinations as recommended | Atherosclerosis | 4.5 | 9.6 | 26.0 | 34.0 | 25.9 |
|  | Stroke | 3.8 | 10.1 | 28.9 | 33.7 | 23.5 |
|  | Myocardial infarction | 3.5 | 9.3 | 26.8 | 35.1 | 25.3 |
|  | CVDs | 3.9 | 9.7 | 27.2 | **34.3** | **24.9** |

Table S5 A sense of self-efficacy, answer to the question: How do you assess your ability to implement this behavior to reduce the risk of the following diseases (n=1000; %)

|  |  | Impossible | Unlikely | Moderately likely | Possible | Very possible |
| --- | --- | --- | --- | --- | --- | --- |
| Healthy diet | Atherosclerosis | 5.0 | 11.9 | 29.1 | 37.3 | 16.7 |
|  | Stroke | 4.4 | 12.5 | 31.1 | 36.5 | 15.5 |
|  | Myocardial infarction | 4.6 | 11.3 | 31.0 | 36.3 | 16.8 |
|  | CVDs | 4.7 | 11.9 | 30.4 | **36.7** | **16.3** |
| Regular physical activity | Atherosclerosis | 4.9 | 12.6 | 29.3 | 33.7 | 19.5 |
|  | Stroke | 4.0 | 13.5 | 30.2 | 31.7 | 20.6 |
|  | Myocardial infarction | 4.5 | 11.2 | 29.9 | 33.0 | 21.4 |
|  | CVDs | 4.5 | 12.4 | 29.8 | **32.8** | **20.5** |
| Avoidance of smoking * | Atherosclerosis | 9.1 | 22.3 | 30.0 | 21.4 | 17.1 |
|  | Stroke | 8.0 | 20.7 | 31.8 | 22.0 | 17.5 |
|  | Myocardial infarction | 7.5 | 21.3 | 30.9 | 22.1 | 18.2 |
|  | CVDs | 8.2 | 21.5 | 30.9 | **21.8** | **17.6** |
| Effective stress management | Atherosclerosis | 5.6 | 14.5 | 31.1 | 35.0 | 13.8 |
|  | Stroke | 5.0 | 13.8 | 32.0 | 33.8 | 15.4 |
|  | Myocardial infarction | 5.4 | 13.6 | 29.4 | 34.8 | 16.8 |
|  | CVDs | 5.4 | 14.0 | 30.8 | **34.5** | **15.3** |
| Medical examinations as recommended | Atherosclerosis | 5.3 | 10.3 | 25.8 | 35.7 | 22.9 |
|  | Stroke | 4.0 | 10.9 | 26.4 | 35.0 | 23.7 |
|  | Myocardial infarction | 4.2 | 9.9 | 26.7 | 35.7 | 23.5 |
|  | CVDs | 4.5 | 10.4 | 26.3 | **35.5** | **23.3** |
| *n=560 | | | | | | |

Table S6 EPPM groups – healthy diet (n=1000; n(%))

| Healthy diet | | total | Indifferent | Proactive | Avoidant | Responsive | Chi2 | p-value |
| --- | --- | --- | --- | --- | --- | --- | --- | --- |
| accommodation | Countryside | 417 (100) | 119 (28.5) | 84 (20.1) | 78 (18.7) | 136 (32.6) | 9.282 | 0.412 |
|  | Town ≤ 200 000 | 290 (100) | 86 (29.7) | 43 (14.8) | 47 (16.2) | 114 (39.3) |  |  |
|  | Town 200 000 -500 000 | 91 (100) | 30 (33.0) | 15 (16.5) | 12 (13.2) | 34 (37.4) |  |  |
|  | Town ≥ 500 000 | 202 (100) | 50 (24.8) | 40 (19.8) | 33 (16.3) | 79 (39.1) |  |  |
| education | Elementary or junior high school | 28 (100) | 9 (32.1) | 4 (14.3) | 7 (25.0) | 8 (28.6) | 21.989 | >0.05 |
|  | Basic vocational | 146 (100) | 60 (41.1) | 19 (13.0) | 22 (15.1) | 45 (30.8) |  |  |
|  | Secondary or post-secondary | 426 (100) | 122 (28.6) | 79 (18.5) | 79 (18.5) | 146 (34.3) |  |  |
|  | Higher education | 400 (100) | 94 (23.5) | 80 (20.0) | 62 (15.5) | 164 (41.0) |  |  |
| employment | Employed (full-time or self-employed) | 817 (100) | 219 (26.8) | 151 (18.5) | 146 (17.9) | 301 (36.8) | 16.302 | 0.178 |
|  | Student | 40 (100) | 12 (30.0) | 9 (22.5) | 7 (17.5) | 12 (30.0) |  |  |
|  | Unemployed | 43 (100) | 21 (48.8) | 6 (14.0) | 6 (14.0) | 10 (23.3) |  |  |
|  | Pensioner/Retiree | 94 (100) | 30 (31.9) | 16 (17.0) | 10 (10.6) | 38 (40.4) |  |  |
|  | Household leader | 6 (100) | 3 (50.0) | 0 (0) | 1 (16.7) | 2 (33.3) |  |  |

Table S7 EPPM groups – regular physical activity (n=1000; n(%))

| Regular physical activity | | total | Indifferent | Proactive | Avoidant | Responsive | Chi2 | p-value |
| --- | --- | --- | --- | --- | --- | --- | --- | --- |
| accommodation | Countryside | 417 | 124 (29.7) | 79 (18.9) | 78 (18.7) | 136 (32.6) | 10.373 | .321  (Ns) |
|  | Town ≤ 200 000 | 290 | 93 (32.1) | 36 (12.4) | 58 (20.0) | 103 (35.5) |  |  |
|  | Town 200 000 -500 000 | 91 | 32 (35.2) | 13 (14.3) | 15 (16.5) | 31 (34.1) |  |  |
|  | Town ≥ 500 000 | 202 | 54 (26.7) | 36 (17.8) | 49 (24.3) | 63 (31.2) |  |  |
| education | Elementary or junior high school | 28 | 9 (32.1) | 4 (14.3) | 7 (25.0) | 8 (28.6) | 24.988 | >0.05 |
|  | Basic vocational | 146 | 65 (44.5) | 14 (9.6) | 26 (17.8) | 41 (28.1) |  |  |
|  | Secondary or post-secondary | 426 | 126 (29.6) | 75 (17.6) | 94 (22.1) | 131 (30.8) |  |  |
|  | Higher education | 400 | 103 (25.8) | 71 (17.8) | 73 (18.3) | 153 (38.3) |  |  |
| employment | Employed (full-time or self-employed) | 817 | 237 (29.0) | 133 (16.3) | 168 (20.6) | 279 (34.1) | 13.089 | .363  (ns) |
|  | Student | 40 | 10 (25.0) | 11 (27.5) | 8 (20.0) | 11 (27.5) |  |  |
|  | Unemployed | 43 | 21 (48.8) | 6 (14.0) | 5 (11.6) | 11 (25.6) |  |  |
|  | Pensioner/Retiree | 94 | 33 (35.1) | 13 (13.8) | 18 (19.1) | 30 (31.9) |  |  |
|  | Household leader | 6 | 2 (33.3) | 1 (16.7) | 1 (16.7) | 2 (33.3) |  |  |

Table S8 EPPM groups – avoidance of smoking (n=560; n(%))

| Avoidance of smoking | | total | Indifferent | Proactive | Avoidant | Responsive | Chi2 | p-value |
| --- | --- | --- | --- | --- | --- | --- | --- | --- |
| accommodation | Countryside | 218 | 76 (34.9) | 38 (17.4) | 32 (14.7) | 72 (33.0) | 10.006 | .350  (ns) |
|  | Town ≤ 200 000 | 165 | 50 (30.3) | 32 (19.4) | 25 (15.2) | 58 (35.2) |  |  |
|  | Town 200 000 -500 000 | 56 | 20 (35.7) | 12 (21.4) | 8 (14.3) | 16 (28.6) |  |  |
|  | Town ≥ 500 000 | 121 | 27 (22.3) | 33 (27.3) | 23 (19.0) | 38 (31.4) |  |  |
| education | Elementary or junior high school | 18 | 6 (33.3) | 4 (22.2) | 3 (16.7) | 5 (27.8) | 12.220 | .201  (ns) |
|  | Basic vocational | 102 | 41 (40.2) | 18 (17.6) | 11 (10.8) | 32 (31.4) |  |  |
|  | Secondary or post-secondary | 243 | 77 (31.7) | 46 (18.9) | 46 (18.9) | 74 (30.5) |  |  |
|  | Higher education | 197 | 49 (24.9) | 47 (23.9) | 28 (14.2) | 73 (37.1) |  |  |
| employment | Employed (full-time or self-employed) | 464 | 134 (28.9) | 97 (20.9) | 77 (16.6) | 156 (33.6) | 24.258 | >0.05 |
|  | Student | 16 | 6 (37.5) | 2 (12.5) | 5 (31.3) | 3 (18.8) |  |  |
|  | Unemployed | 26 | 17 (65.4) | 4 (15.4) | 1 (3.8) | 4 (15.4) |  |  |
|  | Pensioner/Retiree | 49 | 14 (28.6) | 12 (24.5) | 4 (8.2) | 19 (38.8) |  |  |
|  | Household leader | 5 | 2 (40.0) | 0 (0) | 1 (20.0) | 2 (40.0) |  |  |

Table S9 EPPM groups – effective stress management (n=1000; n(%))

| Effective stress management | | total | Indifferent | Proactive | Avoidant | Responsive | Chi2 | p-value |
| --- | --- | --- | --- | --- | --- | --- | --- | --- |
| accommodation | Countryside | 417 | 130 (31.2) | 73 (17.5) | 91 (21.8) | 123 (29.5) | 2.601 | .978  (NS) |
|  | Town ≤ 200 000 | 290 | 82 (28.3) | 47 (16.2) | 68 (23.4) | 93 (32.1) |  |  |
|  | Town 200 000 -500 000 | 91 | 30 (33.0) | 15 (16.5) | 19 (20.9) | 27 (29.7) |  |  |
|  | Town ≥ 500 000 | 202 | 54 (26.7) | 36 (17.8) | 48 (23.8) | 64 (31.7) |  |  |
| education | Elementary or junior high school | 28 | 9 (32.1) | 4 (14.3) | 8 (28.6) | 7 (25.0) | 24.930 | <0.05 |
|  | Basic vocational | 146 | 63 (43.2) | 16 (11.0) | 27 (18.5) | 40 (27.4) |  |  |
|  | Secondary or post-secondary | 426 | 123 (28.9) | 78 (18.3) | 108 (25.4) | 117 (27.5) |  |  |
|  | Higher education | 400 | 101 (25.3) | 73 (18.3) | 83 (20.8) | 143 (35.8) |  |  |
| employment | Employed (full-time or self-employed) | 817 | 234 (28.6) | 136 (16.6) | 198 (24.2) | 249 (30.5) | 18.990 | .089  (NS) |
|  | Student | 40 | 15 (37.5) | 6 (15.0) | 6 (15.0) | 13 (32.5) |  |  |
|  | Unemployed | 43 | 19 (44.2) | 8 (18.6) | 8 (18.6) | 8 (18.6) |  |  |
|  | Pensioner/Retiree | 94 | 25 (26.6) | 21 (22.3) | 12 (12.8) | 36 (38.3) |  |  |
|  | Household leader | 6 | 3 (50.0) | 0 (0) | 2 (33.3) | 1 (16.7) |  |  |

Table S10 EPPM groups – medical examinations as recommended (n=1000; n(%))

| Medical examinations as recommended | | total | Indifferent | Proactive | Avoidant | Responsive | Chi2 | p-value |
| --- | --- | --- | --- | --- | --- | --- | --- | --- |
| accommodation | Countryside | 417 | 131 (31.4) | 72 (17.3) | 73 (17.5) | 141 (33.8) | 14.397 | .109  (NS) |
|  | Town ≤ 200 000 | 290 | 81 (27.9) | 48 (16.6) | 44 (15.2) | 117 (40.3) |  |  |
|  | Town 200 000 -500 000 | 91 | 36 (39.6) | 9 (9.9) | 15 (16.5) | 31 (40.3) |  |  |
|  | Town ≥ 500 000 | 202 | 49 (24.3) | 41 (20.3) | 28 (13.9) | 84 (41.6) |  |  |
| education | Elementary or junior high school | 28 | 11 (39.3) | 2 (7.1) | 5 (17.9) | 10 (35.7) | 25.127 | <0.05 |
|  | Basic vocational | 146 | 62 (42.5) | 17 (11.6) | 21 (14.4) | 46 (31.5) |  |  |
|  | Secondary or post-secondary | 426 | 119 (27.9) | 82 (19.2) | 79 (18.5) | 146 (34.3) |  |  |
|  | Higher education | 400 | 105 (26.3) | 69 (17.3) | 55 (13.8) | 171 (45.8) |  |  |
| employment | Employed (full-time or self-employed) | 817 | 237 (29.0) | 133 (16.3) | 139 (17.0) | 308 (37.7) | 12.934 | .374  (NS) |
|  | Student | 40 | 12 (30.0) | 9 (22.5) | 7 (17.5) | 12 (30.0) |  |  |
|  | Unemployed | 43 | 20 (46.5) | 7 (16.3) | 4 (9.3) | 12 (27.9) |  |  |
|  | Pensioner/Retiree | 94 | 26 (27.7) | 20 (21.3) | 10 (10.6) | 38 (40.4) |  |  |
|  | Household leader | 6 | 2 (33.3) | 1 (16.7) | 0 (0) | 3 (50.0) |  |  |
